# A prospective trial of vaccine to prevent hepatitis B virus reactivation after hematopoietic stem cell transplantation

**DOI:** 10.1038/s41409-020-0833-5

**Published:** 2020-02-18

**Authors:** Koji Nishikawa, Kiminori Kimura, Yoshinobu Kanda, Masaya Sugiyama, Kazuhiko Kakihana, Noriko Doki, Kazuteru Ohashi, Sung Kwan Bae, Kazuhiro Takahashi, Yuko Ishihara, Ishikazu Mizuno, Yasushi Onishi, Masahiro Onozawa, Makoto Onizuka, Masahide Yamamoto, Tetsuya Ishikawa, Kazuaki Inoue, Shigeru Kusumoto, Satoshi Hashino, Hidetsugu Saito, Tatsuya Kanto, Hisashi Sakamaki, Masashi Mizokami

**Affiliations:** 1 Division of Hepatology, Tokyo Metropolitan Cancer and Infectious Diseases Center, Komagome Hospital, Tokyo, Japan; 2 Division of Hematology, Jichi Medical University, Shimotuke, Japan; 3 Genome Medical Science Project, National Center for Global Health and Medicine, Ichikawa, Japan; 4 Division of Hematology, Tokyo Metropolitan Cancer and Infectious Diseases Center, Komagome Hospital, Tokyo, Japan; 5 The Center for Liver Disease, Hamanomachi Hospital, Fukuoka, Japan; 6 Department of Hematology, Hyogo Cancer Center, Akashi, Japan; 7 Department of Hematology and Rheumatology, Tohoku University Hospital, Sendai, Japan; 8 Department of Hematology, Hokkaido University Hospital, Sapporo, Japan; 9 Department of Hematology and Oncology, Tokai University School of Medicine, Isehara, Japan; 10 Department of Hematology, Tokyo Medical and Dental University, Tokyo, Japan; 11 Department of Radiological and Medical Laboratory Sciences, Nagoya University Graduate School of Medicine, Nagoya, Japan; 12 Department of Gastroenterology, Showa University Fujigaoka Hospital, Yokohama, Japan; 13 Department of Hematology and Oncology, Nagoya City University Graduate School of Medical Sciences, Nagoya, Japan; 14 Division of Pharmacotherapeutics, Keio University Faculty of Pharmacy, Tokyo, Japan; 15 The Research Center for Hepatitis and Immunology, National Center for Global Health and Medicine, Ichikawa, Japan

**Keywords:** Clinical trials, Drug development

## Abstract

Hepatitis B virus (HBV) reactivation reportedly occurs frequently after hematopoietic stem cell transplantation (HSCT) in resolved HBV-infected patients. Here, 50 patients with resolved HBV infections and scheduled to undergo HSCT were enrolled; all subjects were vaccinated with three doses of hepatitis B vaccine 12 months after HSCT and the incidence of HBV reactivation was monitored. The patients’ characteristics were: median age, 61 (34–72) years; male/female, 27/19; allogeneic/autologous, 40/6; bone marrow/peripheral blood stem cells/cord blood, 26/16/4. Of the 46 patients who underwent HSCT, 19 were excluded and did not make it to vaccination due to relapse of underlying disease, HBV reactivation within 12 months of HSCT, or transfer of patients. The remaining 27 were vaccinated 12 months after HSCT and monitored for 2 years. Six showed HBV reactivation, with a 2-year cumulative reactivation incidence of 22.2%; the same incidence was 27.3% only in allogeneic HSCT patients. Factors associated with HBV reactivation included the discontinuation of immunosuppressants (*P* = 0.0379) and baseline titers of antibody against hepatitis B surface antigen (*P* = 0.004). HBV reactivation with vaccination following HSCT could occur despite maintenance of serum anti-HBs at more than protective levels.

## Introduction

Hepatitis B virus (HBV) reactivation following anticancer drugs, immunosuppressants, or other chemotherapies is a major clinical problem, irrespective of past infection. HBV reactivation is observed in patients negative for hepatitis B surface antigen (HBsAg) (serum HBV-DNA-negative) and positive for anti-hepatitis B core antigen (HBc) and anti-hepatitis B surface antibodies (anti-HBs) [1]. HBV reactivation is most commonly reported in patients following hematopoietic stem cell transplantation (HSCT) [2–5]. Chronic graft-versus-host disease (GVHD) [2, 6], corticosteroid exposure [2], and loss of anti-HBs [7] are also hypothesized to cause HBV reactivation. In addition, HBV reactivation may result in the discontinuation of effective therapies for hematological diseases, worsening the patient’s prognosis [8]. Current guidelines recommend that HBsAg-negative, anti-HBc-positive patients with undetectable serum HBV-DNA and regardless of anti-HBs status who receive chemotherapy and/or immunosuppression should be followed carefully by alanine aminotransferase (ALT) and HBV-DNA testing and treated with nucleoside/nucleotide analogs (NA) upon confirmation of HBV reactivation before ALT elevation [9, 10]. The frequency of monitoring can range from 1 to 3 months depending on the type of immunosuppressive therapy and comorbidities. NA prophylaxis is recommended for anti-HBc-positive patients receiving bone marrow or stem cell transplantation [5], although the optimal duration of prophylaxis is not known.

The incidence of HBV reactivation after HSCT in HBV-resolved patients is ~40% [11], suggesting that more than half of HSCT patients do not require NA prophylactic administration. Thus, NA prophylaxis seems to be economically questionable. Furthermore, unlike chemotherapy and immunosuppressant administration, the onset of HBV reactivation after HSCT has not been determined, although it has been reported 5 years following HSCT [12]. Such long onset times require long-term preventive NA administration. Considering this, we focused on HB vaccination as an inexpensive and safe alternative, but potentially effective, preventive treatment. We investigated the preventive effect of vaccination, because low anti-HBs titer is associated with reactivation [11, 13]. The European Conference on Infections in Leukaemia recommend that patients infected with HBV before HSCT (HBsAg-negative and anti-HBc-positive) should be assessed regularly for anti-HBs titers and vaccinated if they have unprotective titers; if anti-HBs titers are <10 mIU/mL at 1–2 months after initial three vaccine doses, an additional series of three doses should be considered [14]. However, to the best of our knowledge, no clinical studies have examined the preventive effect of vaccine on reactivation in patients with resolved HBV infection after HSCT. In this study, we investigated the efficacy of HB vaccine in preventing reactivation in resolved HBV-infected patients after HSCT.

## Methods

### Study design and patients

In this multicenter, prospective clinical trial, we sequentially enrolled patients with a past HBV infection, defined as HBsAg-negative and anti-HBc-positive, and scheduled to undergo HSCT between August 1, 2013 and January 31, 2016 at the Komagome Hospital enrollment center. All patients were vaccinated 12 months after HSCT (see Supplementary Materials). The baseline HBV status at enrollment was determined based on serological results for anti-HBs using an anti‐HBs kit and a fully automated chemiluminescent enzyme immunoassay system (Architect i2000SR analyzer; Abbott Japan, Tokyo, Japan). Serologic HBsAg and anti-HBc were measured by chemiluminescent enzyme immunoassay (Fuji Rebio, Tokyo, Japan). Serum HBV-DNA titers were measured using the COBAS TaqMan HBV test kit (Roche, Branchburg, NJ, USA) with a low detection limit of 1.3 log IU/mL.

The review board of each participating institution approved the protocol and all patients provided written informed consent. Eligible participants were between 18 and 75 years of age, scheduled to undergo first HSCT, and were HBsAg-negative and anti-HBc-positive. We excluded patients with human immunodeficiency virus or hepatitis C virus infection. We also excluded patients with a history of past hypersensitivity to vaccination. All eligible patients were vaccinated. The complete eligibility criteria are included in the Supplementary appendix. This study is registered with UMIN (no. UMIN000011543).

### Procedure

HBV-DNA was quantified before HSCT to confirm the patient’s serum as HBV-DNA-negative. Enrolled patients were prospectively monitored by performing the HBV-DNA assay once per month for 1 year after HSCT, and then once every 2 months until 3 years after HSCT. Serum HBsAg and anti-HBs was also monitored once every 2 months. HBV vaccination was commenced 12 months after HSCT, with additional doses administered at 13 and 18 months after HSCT. According to the 2013 IDSA Clinical Practice Guideline [15], three doses of HB vaccine should be administered 6–12 months after HSCT. We postulated that vaccine-induced antibody production was likely to decrease by immunosuppressive treatments; thus, we chose to vaccinate 12 months after HSCT, when chronic GVHD is expected to converge.

### Definition of response to vaccination

Patients were vaccinated with three doses (10 μg) of a recombinant absorbed HBV vaccine (Bimmugen, Kaketsuken, Kumamoto, Japan). Additional inoculations were continued even if anti-HBs titers were elevated after the initial vaccination. Vaccination was performed for both allogeneic and autologous HSCT cases. Vaccine responders were defined as patients in whom (1) anti-HBs titer increased from ≤10 mIU/mL before vaccination to ≥10 mIU/mL after vaccination, or (2) anti-HBs titer was ≥10 mIU/mL before vaccination, but increased in two consecutive assays after vaccination.

### Definition of HBV reactivation

We defined HBV reactivation as conversion from HBV-DNA-negative to HBV-DNA-positive after HSCT, with an HBV-DNA assay threshold of ≥1.3 log IU/mL. When HBV reactivation was observed, therapeutic intervention for chronic hepatitis B (entecavir, 0.5 mg/day) was strongly recommended. In cases of relapse or refractory disease of the primary disease and if a subject was indicated for a second HSCT, the patient was excluded and monitoring was discontinued.

### Safety

Adverse events and reactions observed during the study were evaluated and named using the Common Terminology Criteria for Adverse Events v4.0 Japanese translation, and graded 1–5. Here, deterioration by one or more grades according to the criteria was deemed to be an adverse event. The same criteria were applied for abnormal laboratory values. Acute or chronic GVHD were diagnosed according to the National Institutes of Health consensus guidelines.

### Amino acid sequence of HBs

Serum samples were stored at –80 °C until assayed. DNA was extracted from 200 μL of serum using the QIAamp DNA Blood Mini Kit (Qiagen, Valencia, CA, USA) according to the manufacturer’s instructions, and used for PCR amplification and direct sequencing of the HBV S gene, as previously reported [16, 17]. The sequencing products were analyzed using an ABI 3130*xl* DNA analyzer (Applied Biosystems). The sequences were aligned using the GenBank sequences corresponding to HBV genotypes.

### Outcomes

The primary endpoint was incidence of HBV reactivation in HSCT recipients immunized with HB vaccine. The secondary endpoints were liver toxicity and frequency of fulminant hepatitis caused by HBV reactivation, and safety of HB vaccination. Post hoc exploratory outcomes included HBs escape mutant analysis by sequencing HBsAg using serum of patients showing reactivation after vaccination.

### Statistical analyses

Categorical variables are presented as number (percentage) and continuous variables as median (range). Chi-square or Fisher’s exact tests were used to compare differences between categorical variables. Continuous variables were compared using the Mann–Whitney U test. The cumulative incidence of HBV reactivation was estimated by competing risks model, and risk factors associated with HBV reactivation after vaccination were evaluated using the Fine and Gray model for univariate analysis. We defined censor for any reasons as competing risk. *P* values < 0.05 were considered to be statistically significant. All analyses were performed using R version 3.6.1 (R Foundation for Statistical Computing, Vienna, Austria).

## Results

### Characteristics of the patients

**Fig. 1:**
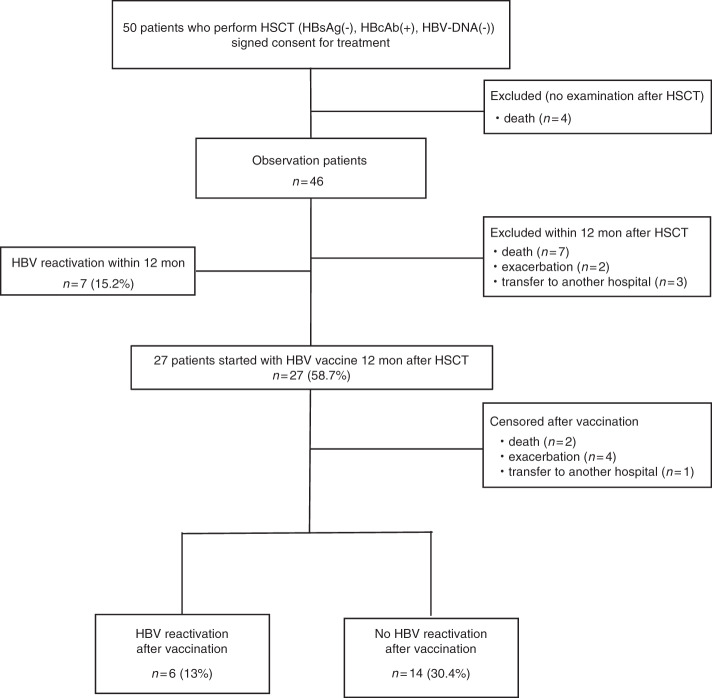
Flow diagram of participants. Trial flow chart.

We enrolled 50 patients who were HBsAg-negative and anti-HBc-positive under HSCT schedule from 11 hospitals in Japan. We observed 46 patients; the other four patients died due to deterioration caused by an underlying disease after HSCT. Patients’ characteristics are summarized in Table 1. The median age was 61 (range 34–72) years, with 27 males (58.7%). Stem cell source was bone marrow for 26 patients (56.5%), peripheral blood stem cells for 16 patients (34.8%), and cord blood (CB) for 4 patients (8.7%). Acute and chronic GVHD occurred in 29 (63%) and 22 (47.8%) patients, respectively. The immunosuppressants used were corticosteroids for 30 (65.2%), cyclosporine for 11 (23.9%), and tacrolimus for 31 (67.4%) patients. During the 12 months of follow-up after HSCT, seven patients died of relapse of an underlying disease, two patients were excluded from further observation due to progression of primary disease, and three patients were transferred to other hospitals; thus, in total, 12 patients were excluded. Seven HBV reactivation events (15.2%) were detected during this period (Fig. 1).

**Table 1:** Patients characteristics, (*n* = 46).

Characteristics	*n* = 46
Age (years), median	61 (34–72)
Male, *n* (%)	27 (58.7%)
Primary disease, *n* (%)	
AML	16 (34.8%)
ALL	4 (8.7%)
CML	2 (4.3%)
MDS	13 (28.2%)
MM	4 (8.7%)
NHL	7 (15.2%)
Stem cell source, *n* (%)	
Bone marrow	26 (56.5%)
PBSC	16 (34.8%)
Autologous/allogeneic	6 (13%)/10 (21.7%)
Cord blood	4 (8.7%)
Conditioning therapy, *n* (%)	
MAC/RIC	23 (50%)/23 (50%)
HLA match, *n* (%)	25 (54.3%)
Immunosuppressant, *n* (%)	
Corticosteroid	30 (65.2%)
Cyclosporine	11 (23.9%)
Tacrolimus	31 (67.4%)
Mycophenolate mofetil	6 (13%)
Short methotrexate	34 (73.9%)
Anti-HBs at baseline, median	100.9 (<2.5–1000<)
Anti-HBc at baseline, median	17.1 (1.2–124)
GVHD, *n* (%)	
Acute GVHD	29 (63%)
Chronic GVHD	22 (47.8%)

### Incidence of HBV reactivation after vaccination

**Fig. 2:**
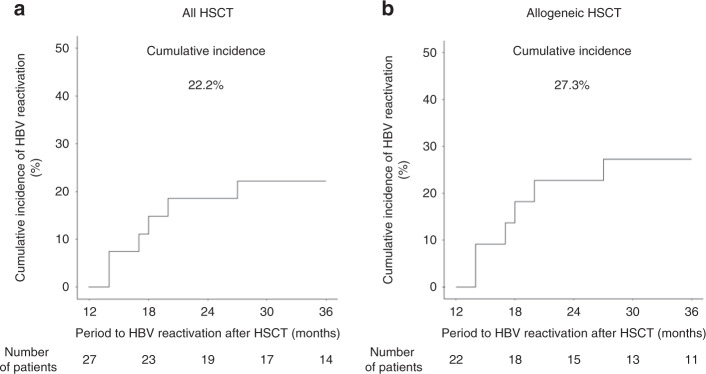
Cumulative incidence of HBV reactivation after vaccination. Cumulative incidence of HBV reactivation after vaccination for (**a**) all HSCT patients and (**b**) allogeneic HSCT patients. The cumulative incidence of HBV reactivation was estimated by competing risks model. We defined censor for any reasons as competing risk. *P* values < 0.05 were considered to be statistically significant.

Excluding the above patients, 27 patients (58.7%) received the first dose of the HB vaccine 12 months after HSCT, with additional doses at 13 and 18 months post-HSCT. These vaccinated patients were observed for 2 years. There were no patients in whom vaccine administration was discontinued due to serious adverse events after vaccination. After vaccination, HBV reactivation occurred in six patients, and the 2-year cumulative incidence of HBV reactivation was 22.2% (Fig. 2a). In case of allogeneic HSCT patients, the 2-year cumulative incidence of HBV reactivation was 27.3% (Fig. 2b). Corticosteroids were used in all HBV-reactivated patients for immunosuppression (Table 2).

**Table 2:** Patients characteristics following HB vaccine.

Characteristics	ALL *n* = 27	HBV reactivation *n* = 6	No HBV reactivation *n* = 21	*P*
Age (years), median	60 (34–72)	59.5 (34–72)	60 (50–68)	0.9665
Male, *n* (%)	15 (55.6%)	5 (83.3%)	10 (47.6%)	0.1819
Primary disease, *n* (%)				
AML	7 (25.9%)	2 (33.3%)	5 (23.8%)	
ALL	4 (14.8%)	1 (16.7%)	3 (14.3%)	
CML	1 (3.7%)	1 (16.7%)	0	
MDS	6 (22.2%)	0	6 (28.6%)	
MM	4 (14.8%)	0	4 (19%)	
NHL	5 (18.5%)	2 (33.3%)	3 (14.3%)	
Stem cell source, *n* (%)				
Bone marrow	15 (55.6%)	5 (83.3%)	10 (47.6%)	0.1819
Autologous PBSC	5 (18.5%)	0	5 (23.8%)	
Allogeneic PBSC	6 (22.2%)	1 (16.7%)	5 (23.8%)	
Cord blood	1 (3.7%)	0	1 (4.8%)	
Conditioning therapy, *n* (%)				
MAC/RIC	16 (59.2%)/11 (40.7%)	2 (33.3%)/4 (66.7%)	14 (66.7%)/7 (33.3%)	0.1874
HLA match, *n* (%)	17 (63%)	4 (66.7%)	13 (61.9%)	0.9999
Acute GVHD, *n* (%)	13 (48.1%)	2 (33.3%)	11 (52.3%)	0.6483
Chronic GVHD, *n* (%)	16 (59.2%)	5 (83.3%)	11 (52.3%)	0.3497
Immunosuppressant, *n* (%)				
Corticosteroid	18 (66.7%)	6 (100%)	12 (57.1%)	0.0707
Cyclosporine	5 (18.5%)	1 (16.7%)	4 (19%)	
Tacrolimus	18 (66.7%)	5 (83.3%)	13 (61.9%)	
Mycophenolate mofetil	3 (11.1%)	1(16.7%)	2 (9.5%)	
Short methotrexate	19 (70.4%)	6 (100%)	13 (61.9%)	
Use immunosuppressant at vaccination	20 (74.1%)	6 (100%)	14 (66.7%)	0.1548
Cessation of immunosuppressant shile observation periods	10 (37%)	0	10 (47.6%)	0.057
Anti-HBs at baseline, median	113 (3–1000<)	47.7 (3.6–273)	172 (3–1000<)	0.0971
Anti-HBs at vaccination, median	34.8 (<2.5–1000<)	11.7 (<2.5–95.3)	37 (<2.5–1000<)	0.1438
Anti-HBc at baseline, median	17.9 (1.8–124)	13.3 (1.8–46.9)	17.9 (2.5–124)	0.3453
HB vaccine response, *n* (%)	10 (37%)	3 (50%)	7 (33.3%)	0.6382
Time to HBV reactivation (months), median	ー	17.5 (14–27)	ー	
HBV-DNA (log IU/mL) at reactivation, median	ー	1.65 (1.4–4)	ー	

The background of patients with HBV reactivation is summarized in Table 3. Reactivation-associated hepatitis did not occur, and no patients showed liver failure owing to entecavir therapy (Table 4). Although Patient 13 showed elevated serum ALT levels, this was due to drug-induced liver damage by voriconazole, and not by HBV reactivation. Within 12 months after HSCT, seven patients (15.2%) had detectable HBV reactivation, consistent with the high rates of reactivation after HSCT described previously [3].

**Table 3:** Characteristics of responders and non-responders to HB vaccination.

	HBV vaccine responder *n* = 10	HBV vaccine non responder *n* = 17	*P*
Age, mean	61 (34–66)	58 (50–72)	0.9115
Male, *n* (%)	3 (30%)	12 (70.6%)	0.0568
Primary disease, *n* (%)			
AML	1 (10%)	6 (35.3%)	
ALL	3 (30%)	1 (5.9%)	
CML	1 (10%)	0	
MDS	0	6 (35.3%)	
MM	2 (20%)	2 (11.8%)	
NHL	3 (30%)	2 (11.8%)	
Stem cell source, *n* (%)			
Bone marrow	4 (40%)	11 (64.7%)	0.2566
Autologous PBSC	3 (30%)	2 (11.8%)	
Allogeneic PBSC	3 (30%)	3 (17.6%)	
CB	0	1 (5.9%)	
Conditioning therapy, *n* (%)			
MAC/RIC	7 (70%)/3 (30%)	9 (52.9%)/8 (47.1%)	0.4475
HLA match, *n* (%)	8 (80%)	9 (52.9%)	0.2305
Acute GVHD, *n* (%)	4 (40%)	9 (52.9%)	0.6946
Chronic GVHD, *n* (%)	6 (60%)	10 (58.8%)	0.9999
Immunosuppressant, *n* (%)			
Corticosteroid	6 (60%)	12 (70.6%)	0.6831
Cyclosporine	3 (30%)	2 (11.8%)	0.3261
Tacrolimus	6 (60%)	12 (70.6%)	0.6831
Mycophenolate mofetil	1 (10%)	2 (11.8%)	0.9999
Short methotrexate	5 (50%)	14 (82.4%)	0.1019
Use immunosuppressant at vaccination	6 (60%)	13 (76.5%)	0.4147
Cessation of immunosuppressant while observation periods	4 (40%)	6 (35.3%)	0.9999

**Table 4:** Characteristics of patients with reactivated HBV after vaccination.

No.	Pt ID	Age	Sex	Disease	Stem cell source	Time to HBV reactivation (month)	HBV-DNA at reactivation (log IU/mL)	HBsAg at reactivation (IU/mL)	HBsAb at reactivation (mIU/mL)	ALT at reactivation (U/L)	HBV genotype	Immunosuppressant at reactivation	cGVHD
1	2	72	M	AML	allo BMT	27	1.6	(−)	60.9	10	N/A	PSL	(+)
2	13	34	M	CML	allo BMT	18	1.7	(−)	39.8	160	C	FK + PSL	(+)
3	17	58	M	NHL	allo BMT	20	1.6	(−)	1.1	21	N/A	FK + PSL	(−)
4	22	64	M	AML	allo BMT	14	1.4	(−)	1.6	23	N/A	FK + PSL	(+)
5	23	61	F	ALL	allo BMT	17	2.3	(−)	881	15	C	FK + PSL	(+)
6	42	52	M	NHL	allo PBSCT	14	4	(+) (2.17)	112	12	C	CyA + PSL	(+)

*AML* acute myeloid leukemia, *CML* chronic myelogenous leukemia, *NHL* non Hodgkin lymphoma, ALL acute lymphocytic leukemia, *BMT* bone marrow transplantation, *PBSCT* peripheral blood stem cell transplantation, *PSL* prednisolone, *FK* tacrolimus, *CyA* cyclosporin.

### Factors associated with reactivation

**Fig. 3:**
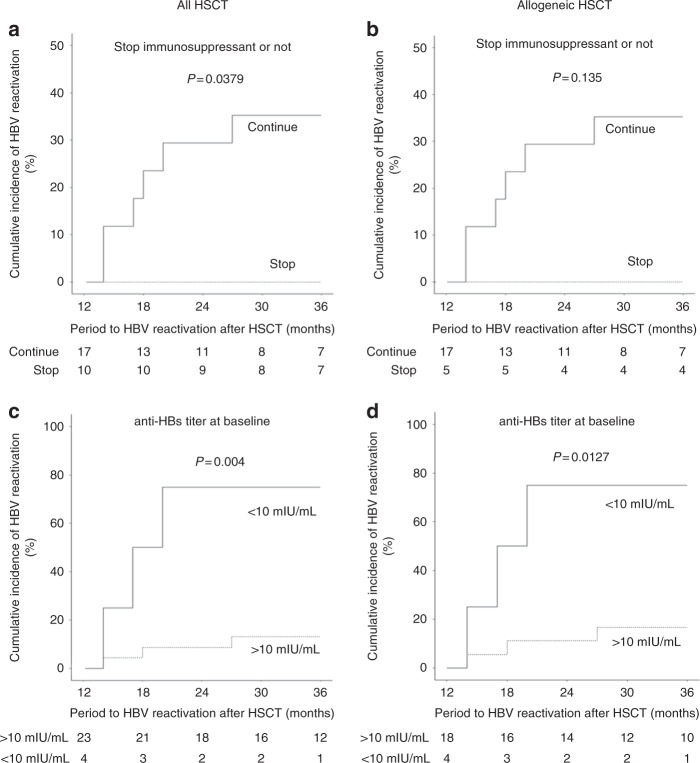
Factors associated with HBV reactivation after vaccination. Factors associated with HBV reactivation after vaccination for (**a**) all HSCT patients with or without cessation of immunosuppressant, (**b**) allogeneic HSCT patients with or without cessation of immunosuppressant, (**c**) all HSCT patients with anti-HBs titer <10 mIU/mL or >10 mIU/mL at baseline, and (**d**) allogeneic HSCT patients with anti-HBs titer <10 mIU/mL or >10 mIU/mL at baseline. Risk factors associated with HBV reactivation after vaccination were evaluated using the Fine and Gray model for univariate analysis. We defined censor for any reasons as competing risk. *P* values < 0.05 were considered to be statistically significant.

The discontinuation of immunosuppressants and anti-HBs titers at baseline was identified to be most closely associated with the cumulative incidence of HBV reactivation (Fig. 3a–d). Interestingly, chronic GVHD related to the use of immunosuppressants did not contribute significantly to the incidence of HBV reactivation. Furthermore, anti-HBs titers at the time of vaccination were not correlated with HBV reactivation, unlike baseline titers.

### Response to HBV vaccine

**Fig. 4:**
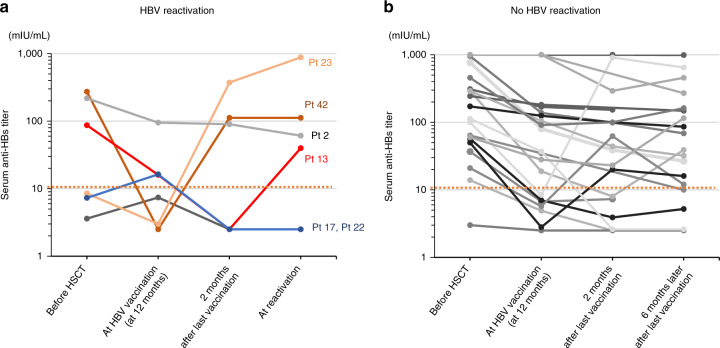
Changes in serum anti-HBs titer after vaccination. Changes in serum anti-HBs are shown before HSCT, after the first vaccination, 2 or 6 months after final vaccination for (**a**) HBV-reactivated and (**b**) no reactivated patients.

As shown in Fig. 4, no significant difference was found in the incidence of HBV reactivation between the vaccine responder and nonresponder groups. Among the 21 patients whose infections did not reactivate, 16 (76.2%) had anti-HBs titers of 10 mIU/mL or more. In contrast, five patients (23.8%) did not show reactivated infection, despite anti-HBs titers of <10 mIU/mL. However, we found high levels of serum anti-HBs (>10 mIU/mL) in four (66.7%) of six patients with HBV reactivation, suggesting escape mutants of HBsAg. To test this hypothesis, HBsAg sequencing was performed using sera from all HBV-reactivated patients.

### Amino acid sequence of HBsAg and clinical course of patients with HBV reactivation following HB vaccination

**Fig. 5:**
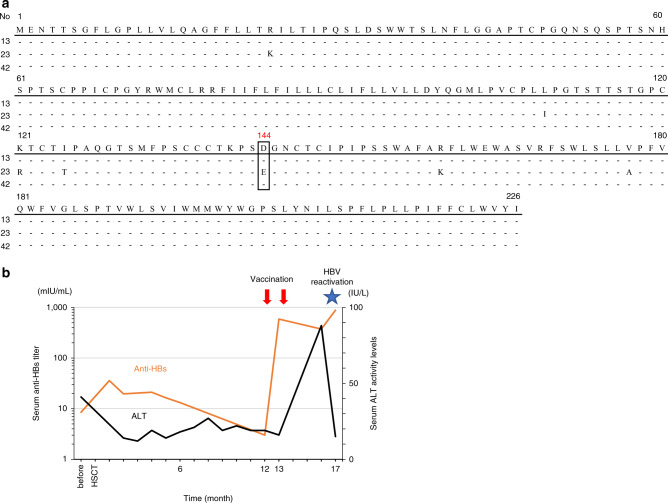
Amino acid sequences of hepatitis B surface antigen and clinical course of patient 23 (Pt 23). **a** Sequence alignments with consensus (top line) for three patients (13, 23, and 42) who exhibited HBV reactivation after HB vaccination. Only residues that were different from the consensus sequence are shown. **b** Clinical course of reactivated HBV infection for Pt 23, with an HBsAg escape mutation after HB vaccination. Orange lines indicate serum anti-HBs titer levels, and black lines indicate serum alanine aminotransferase levels. Red arrows indicate vaccination time points.

Sequencing of the HBsAg gene in the HBV-reactivated patients’ sera was successful in only three (patients 13, 23, and 42) out of six cases (Table 3), because HBV-DNA levels were very low in the sera of the remaining three cases. In addition, whenever HBV reactivation was confirmed, entecavir treatment was immediately initiated, reducing viremia and making HBsAg sequencing impossible in the remaining three cases. The three tested patients presented HBsAg mutations that included sL110I, sK122R, sD144E, and sR160K, and sV177A only in one case (patient 23) (Fig. 5). Among these mutations, the sD144E substitution, which resides in a major hydrophilic region of S-HBsAg [18, 19], is known to hamper S-HBsAg recognition by antibodies. Thus, we observed only one HBV reactivation that could be attributed to HBsAg escape mutation.

## Discussion

In our study, the 2-year cumulative incidence of reactivation was 22.2% after vaccine administration, and 28.9% in 3 years after HSCT despite vaccine intervention. This suggests that HB vaccination cannot prevent HBV reactivation completely; this is in contrast to a previous study reporting preventive effects of vaccination retrospectively [20]. Seto et al., in a prospective study, reported an HBV reactivation incidence of ~40%, 2 years after HSCT [11]. Although further randomized studies are needed, the effectiveness of vaccination is currently questionable. Generally, waiting 6–12 months after HSCT is not considered a major risk for HBs-seronegative patients before HSCT administration [21]. Reportedly, HB vaccination can be administered from 6 to 12 months following HSCT, but early posttransplant vaccination can be considered for anti-HBs-positive patients before HSCT [21]. Although we chose vaccination time at 12 months after HSCT, others have reported that higher titers of anti-HBs produce higher reactivity to the vaccine [20, 22]; therefore, one should consider vaccine administration at 6 months following HSCT.

Anti-HBs titers are negatively correlated with HBV reactivation as reported in some retrospective studies [1, 3, 23]. Therefore, we vaccinated our subjects to elevate anti-HBs titers. However, four patients, in whom reactivation occurred despite high anti-HBs titers (>10 mIU/mL), were considered to have minimal protective-antibody titers (Fig. 4).

Patients with immunosuppression-associated HBV reactivation reportedly show a high degree of genetic complexity in the S-HBsAg sequences in their viral isolates, suggesting that HBsAg mutations may cause reactivation [18]. HB vaccination reportedly selects HBsAg escape mutations under immunosuppressed conditions; therefore, we performed HBsAg sequencing after HB vaccination to examine the relationship between reactivation and escape mutations. Induction of HBsAg escape mutants after liver transplantation [24] and HSCT [25, 26] have been reported principally in two clinical settings: vaccinated newborns of HBV-infected mothers and recipients of orthotopic liver transplant receiving human monoclonal anti-HBs or hyperimmune HBV immunoglobulins [27, 28]. Amino acid substitutions within the “a” determinant of HBsAg can lead to conformational alterations that affect binding of neutralizing antibodies. The most frequent substitutions include sG145A and sD144E, although many other substitutions have been described in the HBsAg outside of the “a” determinant sequence and in the pre-S region [29]. Here, only a single-HBsAg escape mutant (sD144E) was detected, with sL110I, sK122R, sD144E, and sR160K substitutions also present in the major hydrophobic region. Notably, no escape mutants were detected in patient 42 and patient 13, who had serum anti-HBs titers >10 mIU/mL (112 and 39.8 mIU/mL, respectively). This suggests the importance of both cell-mediated and humoral immunity for controlling HBV. In fact, anti-HBs titers were 10 mIU/mL or less in four patients without HBV reactivation, and we consider that T cells were controlling HBV in these patients (Fig. 4). Nonetheless, vaccination is likely to select for escape mutations in the HBsAg gene during immunosuppressive treatment following HSCT; therefore, such cases warrant careful monitoring.

Recently, the response (anti-HBs titers of >10 mIU/mL) to HB vaccine in normal healthy volunteers was reported to be 90% [30]. Furthermore, 64% of post-HSCT patients seroconverted to anti-HBs-positive [31]. Considering these reports, it appears that our 37% (10/27) response to vaccination is low. The anti-HBc-positive patients were past HBV-infected patients and would be expected to have a strong secondary immune response to vaccination [32]. Two possible reasons may explain the low response rate to vaccination. Firstly, at 12 months after HSCT, almost all recipients’ T and B cells have been replaced with donor-derived cells [33], suggesting presence of only few recipient-derived memory T and B cells. If donor is an HBV-resolved infection case (anti-HBc positive), it may contribute to anti-HBs production via memory T cells after HSCT [34–36]. Unfortunately, the donor for most patients with HBV reactivation was from a bone marrow bank, and it was not possible to analyze whether donor-derived HBV infection affected reactivation. Secondly, the host ability to produce antibody is reduced by immunosuppressive therapies. A recent report indicated HLA-DR-DQ and butyrophilin-like protein 2 to be important in response to HB vaccine [30], and future studies are necessary to better understand these host factors.

Discontinuation of immunosuppressive treatment was associated with HBV reactivation after vaccination. Although immunosuppressants are usually used for prevention and treatment of chronic GVHD, they have reportedly been implicated in HBV reactivation [11]. Among the immunosuppressants, use of corticosteroids was nearly significantly (*P* = 0.056) associated with reactivation. Corticosteroids can act synergistically to promote viral replication through direct transcriptional regulation of the HBV genome [37]; thus, discontinuation of steroid treatment may be an important factor for preventing reactivation.

HBV reactivation-associated liver disease (de novo hepatitis) after allogeneic HSCT is often temporary and not serious, although fatal liver damage has been occasionally reported [38, 39]. In the 13 patients with HBV reactivation in this study, most showed no biochemical parameters of liver damage. In addition, no patient developed severe liver failure owing to antiviral treatment at onset of HBV reactivation. As previously reported [11, 40], in HSCT that causes frequent reactivation, periodic monitoring of HBV-DNA facilitates timely therapeutic intervention to prevent serious liver damage. Furthermore, 15% patients with HBV reactivation were identified within 12 months after HSCT in this study. For these very early reactivation cases, it is important to prevent reactivation by NA prophylaxis. We propose that cases where the anti-HBs titer at the time of HSCT is low (10 mIU/mL or less) should be considered for prophylactic administration with a focus on treatment target.

Cost-effectiveness is a very important issue, and in the current study, medical expenses were calculated for Japan. The price of lamivudine drug is 484.7 yen/100 mg, amounting to ~15000 yen a month if taken once a day. In contrast, HBV-DNA testing costs about 2800 yen per month. Since HBV-DNA testing costs are lower, we consider NA administration economical when reactivated by HBV-DNA monitoring every 1–3 months. However, as with the preventive administration of lamivudine, the question is how long it will be necessary to measure HBV-DNA.

In conclusion, post-HSCT HBV reactivation was found to occur at a constant rate after vaccination. However, no reactivation was observed in patients in whom immunosuppressant administration was discontinued after vaccination. Our findings suggest that HBV reactivation depends on the host’s immune function. As one of the three tested HBV-reactivated patients showed an HBsAg escape mutation following vaccination, vaccination could be a factor inducing HBsAg escape mutation. After HSCT for resolved HBV-infected patients, preventive administration of antiviral drugs was tested; however, increasing the number of tested patients is necessary for greater statistical power and better assessing the efficacy, safety, and cost-effectiveness of the vaccination.

## Supplementary information

Supplementary materials
