# Supplementary material for: A prospective trial of vaccine to prevent hepatitis B virus reactivation after hematopoietic stem cell transplantation
Source: Bone Marrow Transplant. 2020 Feb 18;55(7):1388–98. doi: 10.1038/s41409-020-0833-5 (PMC7329632; doi:10.1038/s41409-020-0833-5)
Supplement: Supplementary file 1 — Supplementary materials [file 41409_2020_833_MOESM1_ESM.docx]

**Inclusion and exclusion criteria**

Patients who meet all the inclusion criteria and none of the exclusion criteria below are eligible for enrollment.

Inclusion criteria

1. Scheduled to undergo first HSCT
2. Both HBsAg-negative and HBcAb-positive
3. Serum bilirubin ≤2.0 mg/dL
4. AST and ALT both ≤2.5 times the upper limit of the laboratory reference range
5. Serum creatinine ≤1.5 times the upper limit of the laboratory reference range
6. PaO_2_ ≥60 torr or SpO_2_ ≥90% in room air before treatment
7. Performance Status of 0–2
8. ≥20 and ≤75 years-of-age
9. Written consent to participate in this study has been given voluntarily by the patient.

Exclusion criteria

1. HCV antibody-positive
2. HIV antibody-positive
3. Past hypersensitivity to vaccination
4. Past hypersensitivity to a biological preparation
5. Patient has a serious infectious disease (general condition is judged markedly poor due to infectious disease, such as due to septic shock)
6. Receiving dialysis or introduction of dialysis is being considered
7. Patient has a psychiatric disorder that requires treatment
8. Patient has poorly controlled diabetes mellitus or hypertension, or has heart failure
9. Pregnant or nursing
10. Patient is judged ineligible for this study by an investigator
